# Supplementary material for: CO2 Breathing Prior to Simulated Diving Increases Decompression Sickness Risk in a Mouse Model: The Microbiota Trail Is Not Forgotten
Source: Int J Environ Res Public Health. 2024 Aug 28;21(9):1141. doi: 10.3390/ijerph21091141 (PMC11431549; doi:10.3390/ijerph21091141)
Supplement: Supplementary file 1 [file ijerph-21-01141-s001.zip › ijerph-3116667-supplementary.pdf]

Supplementary data

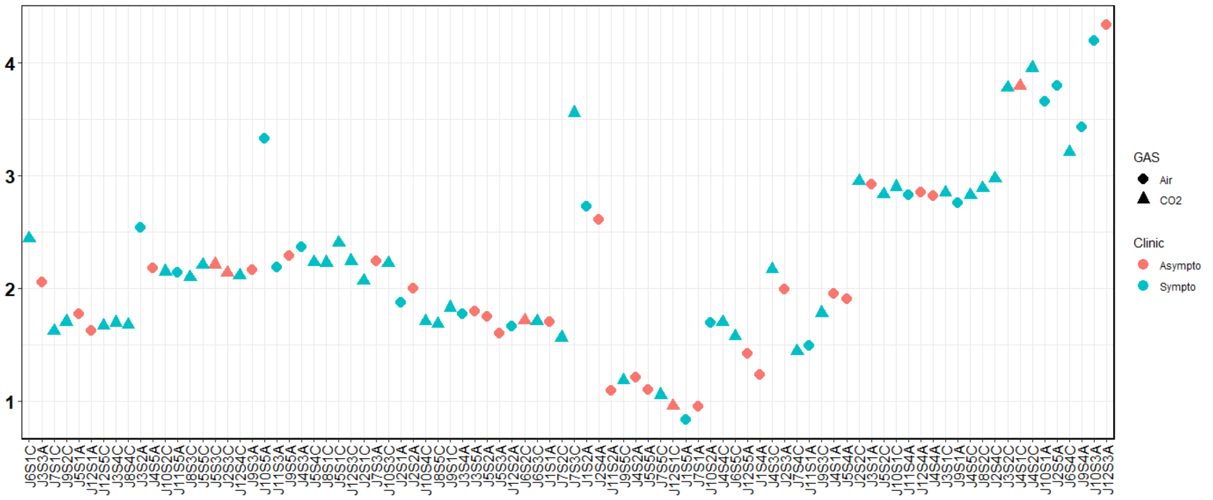

Figure S1. Distribution of Shannon diversity of microbial communities obtained with the 16 rDNA dataset. The colors represent the clinical group and the shape represents the gas group.

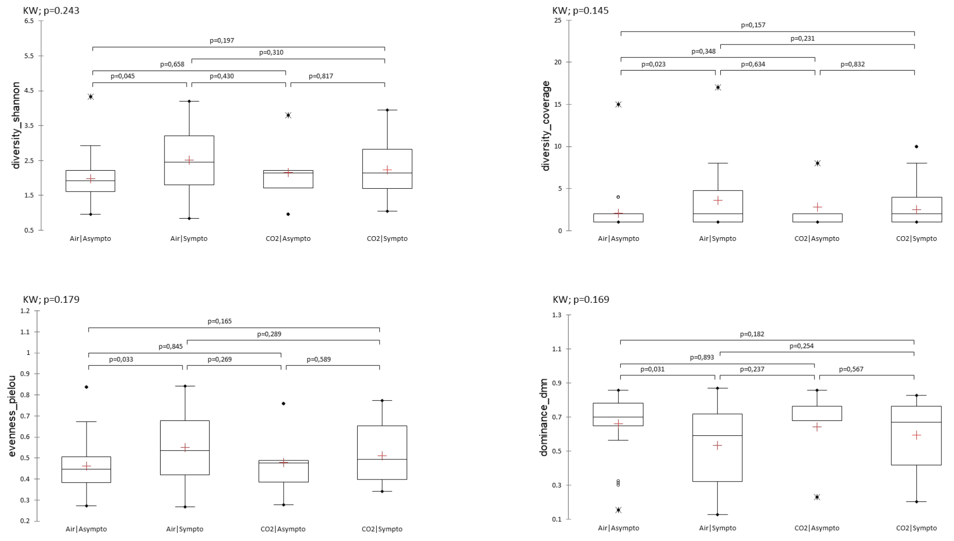

Figure S2 Diversity index. Kruskal-Wallis (KW) analysis with post-hoc dunn test (Bonferroni correction: 0,0083).

Genus	Raw Abondances				Relatives Abondances				
	KW	Post-hoc p<0.05	Post-hoc p<0.0083	Bilan I	KW	Post-hoc p<0.05	Post-hoc p<0.0083	Bilan II	Bilan III
Allorhizobium....	0.072	Yes	No	Asym_Air vs Asym_CO2<br>Sym_Air vs Asym_CO2<br>Sym_CO2 vs Asym_CO2	0.053	Yes	Yes	**Asym_Air vs Asym_CO2**<br>Sym_Air vs Asym_CO2<br>Sym_CO2 vs Asym_CO2	**Asym_Air vs Asym_CO2**<br>Sym_Air vs Asym_CO2<br>Sym_CO2 vs Asym_CO2
Eub Nodatus gp	0.186	No	No		0.136	Yes	No	Asym_Air vs Sym_Air	
Cand Arthro	0.201	No	No		0.235	Yes	No	Asym_Air vs Sym_Air	
Anaerostripes	0.136	Yes	No	Asym_Air vs Asym_CO2	0.097	Yes	No	Asym_Air vs Asym_CO2	Asym_Air vs Asym_CO2
Blautia	**0.002**	Yes	**Yes**	Asym_Air vs Sym_CO2<br>**Sym_Air vs Sym_CO2**	**0.001**	Yes	**Yes**	Asym_Air vs Sym_Air<br>Asym_Air vs Sym_CO2<br>**Sym_Air vs Sym_CO2**	Asym_Air vs Sym_CO2<br>**Sym_Air vs Sym_CO2**
GCA-900066575	**0.045**	Yes	**Yes**	**Asym_Air vs Sym_Air**	**0.041**	Yes	**Yes**	**Asym_Air vs Sym_Air**	**Asym_Air vs Sym_Air**
Roseburia	0.071	Yes	No	Asym_Air vs Sym_Air<br>Sym_Air vs Sym_CO2	0.057	Yes	No	Asym_Air vs Sym_Air<br>Sym_Air vs Sym_CO2	Asym_Air vs Sym_Air<br>Sym_Air vs Sym_CO2
Nostoc	0.086	Yes	No	Asym_Air vs Sym_Air	0.111	Yes	No	Asym_Air vs Sym_Air	Asym_Air vs Sym_Air
Tolypothrix	0.294	No	No		0.257	Yes	No	Asym_Air vs Asym_CO2	
Candidatus Sacch	**0.043**	Yes	No	Asym_Air vs Sym_Air	**0.045**	Yes	No	Asym_Air vs Sym_Air	Asym_Air vs Sym_Air

Table S1 Relative and raw abundances. Summary table according to Kruskal-Wallis (KW) analysis with post-hoc dunn test (Bonferroni correction: 0,0083).
